# Intraarticular overexpression of Smad7 ameliorates experimental arthritis

**DOI:** 10.1038/srep35163

**Published:** 2016-10-12

**Authors:** Shih-Yao Chen, Ai-Li Shiau, Chao-Liang Wu, Chrong-Reen Wang

**Affiliations:** 1 Section of Rheumatology, Department of Internal Medicine, National Cheng Kung University Hospital, No. 138, Sheng-Li Road, Tainan, 70428, Taiwan; 2 Department of Microbiology and Immunology, National Cheng Kung University Medical College, Tainan, 70101, Taiwan.; 3 Department of Biochemistry and Molecular Biology, National Cheng Kung University Medical College, Tainan, 70101, Taiwan

## Abstract

Rheumatoid arthritis (RA) and Crohn’s disease (CD) are autoimmune disorders with a crosstalk between their pathogenesis such as increased expression of TNF in the target organs. Despite a successful clinical trial with an oral Smad7 antisense oligonucleotide in CD, intraarticular (i.a.) modulation of Smad7 expression has not been performed in rheumatoid joint yet. In this study, contradictory to the findings in CD mucosa, higher levels of pSmad2/3 were found in RA synovium. *In vitro* experiments with synovial fibroblasts revealed that higher acetylated Smad7 expression was associated with lower activation status. Abundant expression of synovial pSmad2/3 with increased levels during the progression of arthritis was detected in collagen-induced arthritis (CIA) mice. To prove the concept that overexpressing Smad7 as a therapeutic strategy in rheumatoid joint, the i.a. injection of lentiviral vectors carrying Smad7 (LVSmad7) was carried out in CIA mice. In LVSmad7-injected joints, there were lower arthritis and histological scores with less synovitis, synovial hyperplasia and erosion on cartilage and bone as well as reduced IL-17 and TNF expression levels in comparison with other control groups. In conclusion, we demonstrate that lentiviral vector-mediated i.a. overexpression of Smad7 can ameliorate rheumatoid joint, implicating a pharmacological development of Smad7-based molecular strategy in RA.

The binding of transforming growth factor (TGF)-β to its receptors can phosphorylate the second messenger complex Smad2/3, recruit Smad4 into a complex with further translocation into the nucleus where it regulates specific gene expression responses by binding to the promoters together with other transcription factors^1^,^2^. Smad7, a negative regulator of TGF-β signaling by interfering with the Smad2/3 phosphorylation and through the crosstalk with other signaling pathways, is involved in miscellaneous disease states, with the implication as a therapeutic target^3^,^4^. Despite a safety concern for fibrosis and stenosis of the bowel due to the overactivated TGF-β signaling^5^, there was a successful trial in Crohn’s disease (CD) with an oral Smad7 antisense oligonucleotide to block its local production^6^. TGF-β is expressed by cellular populations like synovial fibroblasts (SFs), and its functional role has been extensively studied in various genetically modified mice and by direct injection of TGF-β or antibodies against this molecule or its receptor in experimental arthritis models^7^. Nevertheless, intraarticular (i.a.) modulation of Smad7 molecule has not been performed in rheumatoid joint yet. Herein, we examined the therapeutic effects of lentiviral vector-mediated overexpressing Smad7 molecule in collagen-induced arthritis (CIA) joints. Synovial tissues were obtained from rheumatoid arthritis (RA) and osteoarthritis (OA) patients or CIA and normal mice with further purification of SFs for *in vitro* experiments.

## Results

### Increased expression levels of pSmad2/3 and histone deacetylase 1 (HDAC1) in RA synovial tissues

**Figure 1:**
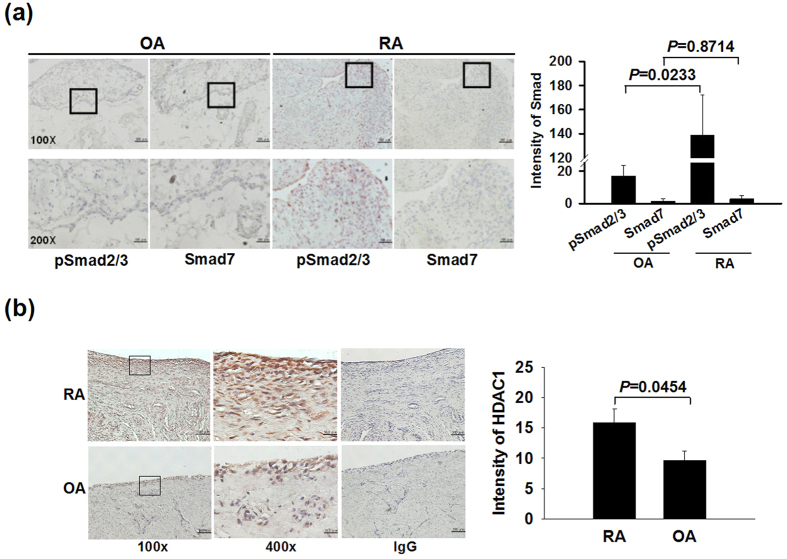
Increased expression levels of pSmad2/3 and HDAC1 in RA synovial tissues. Representative immunohistochemical images and quantitative analysis of pSmad2/3, Smad7 (**a**) and HDAC1 (**b**) synovial expression in RA and OA patients (5 patients per group). Photographic scale bars at 100 × ( = 100 μm), 200 × ( = 50 μm), 400 × ( = 25 μm), and IgG ( = 100 μm) magnifications. Boxed areas including synovial lining and sublining layers are shown at higher magnification in the panels beneath them. Values are mean ± SEM. Results are representative of 2 independent experiments with similar results.

At first, the expression of pSmad2/3 and Smad7 were analyzed in synovial tissues from arthritis patients. There were higher levels of pSmad2/3 in RA patients as compared with OA counterparts, while Smad7 levels were scarcely detected (Fig. 1a). Furthermore, synovial expression levels of HDAC1, a known Smad7 regulator able to reduce its protein stability through deacetylation^8^, were higher in RA in comparison with OA patients (Fig. 1b).

### Increased expression levels of pSmad2/3 in CIA synovial tissues

**Figure 2:**
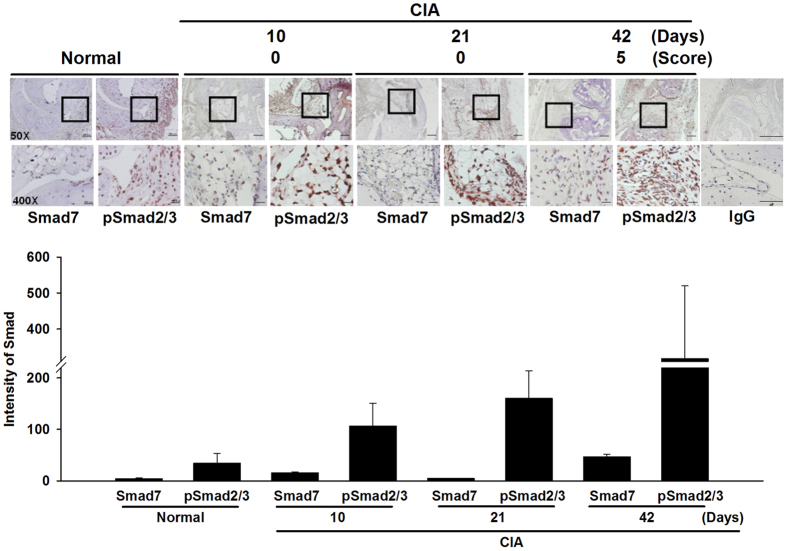
Increased expression levels of pSmad2/3 in CIA synovial tissues. Representative immunohistochemical images and quantitative analysis of pSmad2/3 and Smad7 synovial expression in CIA and normal mice (3 mice per time point). Photographic scale bars at 50 × ( = 200 μm), 400 × ( = 25 μm), and IgG (50 ×  = 600 μm, 400 ×  = 75 μm) magnifications. Boxed areas including synovial lining and sublining layers are shown at higher magnification in the panels beneath them. Values are mean ± SEM. Results are representative of 2 independent experiments with similar results.

Next, we examined an experimental arthritis model for synovial expression of pSmad2/3 and Smad7. There were abundant expression of synovial pSmad2/3 with increased levels from day 10 onward during the progression of CIA, and lower Smad7 levels with a similar kinetic pattern except a decline on day 21 (Fig. 2), suggesting an upregulated TGF-β signaling activity in rheumatoid joint.

### Higher expression levels of acetylated Smad7 associated with lower activation status in SFs

**Figure 3:**
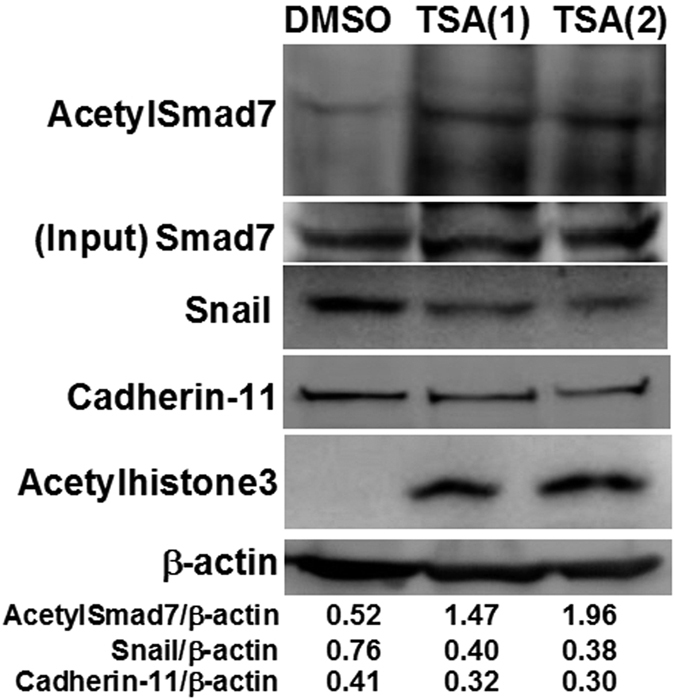
Higher expression levels of acetylated Smad7 associated with lower activation status in SFs. Representative immunoblots of acetylSmad7, Smad7, Snail, Cadherin-11, acetylhistone3 and β-actin expression in 1 or 2 μM TSA- and DMSO-treated cultured SFs. Results are representative of 3 independent experiments with similar results.

Furthermore, *in vitro* experiments were carried out by culturing SFs. With the presence of HDAC1 inhibitor trichostatin A (TSA) in the SFs culture for 24 hr, there was a dose-dependent increase in acetylation levels of Smad7 (Fig. 3a). Notably, higher acetylated Smad7 levels were associated with down-regulated expression levels of Snail, a TGF-β-inducible transcription factor capable of activating SFs to perpetuate the RA activity^9^. In addition, the phosphorylated nuclear factor (NF)-κB p65 signaling intensities of cultured SFs were reduced in the presence of TSA (data not shown). Collectively, together with the finding of higher synovial expression levels of HDAC1, these results suggest that overexpressing Smad7 to down-regulate the TGF-β signaling can be a beneficent approach in the RA therapy.

### Amelioration of CIA by i.a. overexpression of Smad7 with reduced synovial IL-17 and TNF expression

**Figure 4:**
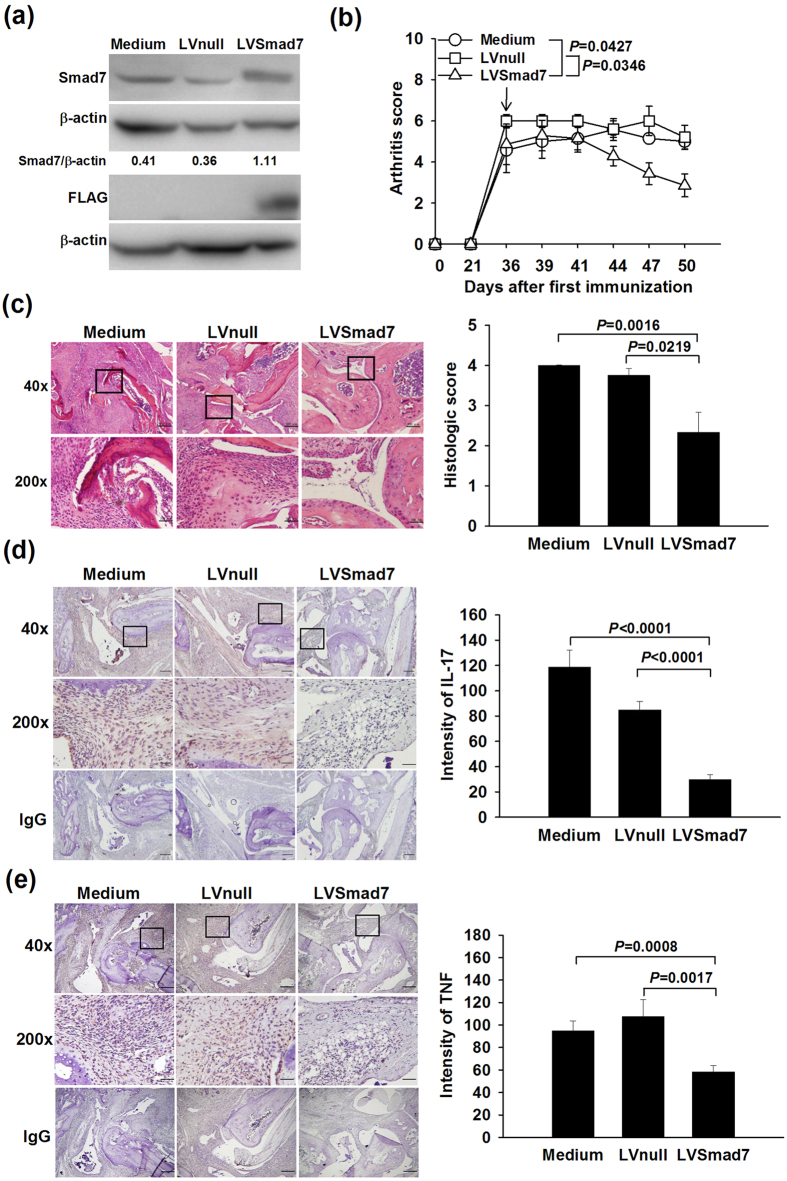
Amelioration of CIA by i.a. overexpressing Smad7 with reduced synovial IL-17 and TNF expression. Representative immunoblots of Smad7, Flag and β-actin expression in LVSmad7-, LVnull- and medium-treated synovium (pooled samples with 3 joints) **(a)**. CIA joints receiving the LVSmad7 injection had lower arthritis scores (10 joints per group) **(b)**. Arrow indicates the injection time. Representative H&E-stained photographs of CIA joints receiving different treatments and histological scores (5 joints per group) **(c)**. Representative immunohistochemical images and quantitative analysis of IL-17 **(d)** and TNF **(e)** expression in LVSmad7-, LVnull- and medium-treated synovium (5 joints per group). Synovial sections for histopathological examinations were obtained by cutting the mouse ankles on the sagittal plane through the center of the joint line. Photographic scale bars at 40 × ( = 250 μm), 200 × ( = 50 μm), and IgG ( = 250 μm) magnifications. Boxed areas are shown at higher magnification in the panels beneath them. Values are mean ± SEM. Results are representative of 2 independent experiments with similar results.

The efficacy of overexpressing Smad7 in CIA joints was verified by analyzing the expression levels of Smad7 and Flag in LVSmad7, LVnull and medium alone-treated synovial tissues with the immunoblot assay. In Fig. 4a, there were significantly increased levels of Smad7 with the presence of Flag in LVSmad7-injected synovial tissues. In order to prove the concept that overexpressing Smad7 as a therapeutic strategy in rheumatoid joint, we performed the i.a. injection of lentiviral vectors in CIA mice during the progression of arthritis (all groups with 100% incidence in this study). There were lower arthritis and histological scores with less synovitis, synovial hyperplasia and erosion on cartilage and bone in LVSmad7-injected joints in comparison with other control groups (Fig. 4b,c). Furthermore, LVSmad7-treated synovial tissues had reduced IL-17 and TNF expression levels as compared with LVnull or medium alone-treated synovium (Fig. 4d,e); however, there were no differences in the IL-6 expression levels among three treatment groups (data not shown), suggesting that the activation of non-canonical TGF-β-NF-κB cross-talk is in favor of the TNF-induced NF-κB signaling pathway in rheumatoid joint^10^.

## Discussion

RA and CD are organ-specific autoimmune disorders with a crosstalk between their pathogenesis such as increased expression of TNF in their target organs^6^,^11^. In particular, together with IL-6 through the activation of STAT3, TGF-β can induce RORγt to orchestrate the IL-17 expression in naïve CD4-positive T cells, resulting in the differentiation into Th17 cells, a therapeutic target under active pharmacological development in both diseases^12^,^13^. Indeed, in this study, interfering with the TGF-β signaling could reduce synovial IL-17 and TNF expression levels. Notably, there are conflicting results regarding the expression levels of pSmad2/3 with up-regulation in RA synovium and down-regulation in CD mucosa, suggesting that different regulatory mechanisms exist in the TGF-β signaling pathway of two distinct autoimmunity status^7^,^14^. Consequently, contradictory approach has been used by overexpressing Smad7 and silencing this molecule in rheumatoid joint and inflamed bowel, respectively, with therapeutic responses in both diseases.

Interestingly, Smad7 participates in the crosstalk between TGF-β and diverse signaling pathways^3^. Inhibition of NF-κB activation by Smad7 can be mediated through the binding to IRAK1 and TAB2/TAB3 to block IL-1R and TNFR signaling, respectively, resulting in lower levels of pro-inflammatory cytokines and less anti-apoptotic signaling activity^3^,^15^,^16^. Notably, in tumor cells, a cross-talk has been identified between TGF-β and NF-κB signaling pathways mediated through TAK1 and Smad7^10^. Indeed, in this study, there was a decrease in the signaling intensities of phosphorylated NF-κB p65 in SFs, raising a possibility that such a cross-talk is mediated through Smad7 in the SFs from rheumatoid joint. Moreover, Smad7 can reduce the Wnt signaling, a pathogenic pathway up-regulated by TNF in RA, through the complex formation with β-catenin and Smurf2 with degradation of the former molecule via the action of proteasome^3^,^9^,^17^. Thus, in addition to the TGF-β-dependent signaling, Smad7 can modulate other pathogenesis-related signaling pathways to ameliorate rheumatoid joint.

In conclusion, we demonstrate that lentiviral vector-mediated i.a. overexpression of Smad7 can ameliorate rheumatoid joint, implicating a pharmacological development of Smad7-based molecular strategy in RA.

## Methods

### Ethics statement

The Institutional Review Board of National Cheng Kung University Hospital approved the permission to obtain human synovial specimens, and informed consent was obtained from all subjects. The Institutional Animal Care and Use Committee of National Cheng Kung University approved the animal experiments. All methods relating to humans were performed in accordance with the relevant guidelines and regulations, and were approved by the Institutional Review Board of National Cheng Kung University Hospital. All animal experiments were conducted in accordance with the approved institutional guidelines.

### Immunohistochemical analysis

Paraffin-embedded synovial sections were processed and stained with anti-Smad7 (R&D systems), anti-phosphorylated Smad2/3 (Santa Cruz), anti-histone deacetylase 1 (HDAC1, Santa Cruz), or isotype control IgG (Santa Cruz), followed by secondary antibody and substrate chromogen, and their averaged expression intensities in 5 blindly chosen random fields were quantified with HistoQuest analysis software (TissueGnostics), as previously described^9^,^18^.

### Construction and production of lentiviral plasmids

The pCMV-Tag2B plasmid (Addgene) was digested with *Nde*I and *Eco*RI to release the CMV immearly promoter with N-terminal Flag, subcloned into pCMV5-Smad7-HA (Addgene), resulting in pCMV-Flag-Smad7-HA. It was further excised and subcloned into the lentiviral plasmid pLKO.1-shLuc (National RNAi Core Facility, Academia Sinica, Taiwan) by digestion with *Nde*I and *Xba*I to generate pLKO.1-Flag-Smad7-HA. The control plasmid pWPXL-null encoding no transgene was constructed from the pWPXL by digestion with *Pme*I and *Eco*RI to delete the GFP cDNA. Recombinant lentiviral vectors were produced by transfecting 293T cells with pLKO.1-Flag-Smad7-HA or pWPXL-null, along with packaging plasmids psPAX2 and envelope plasmids pMD2.G by using the calcium phosphate precipitation method, and LVSmad7 and LVnull vectors were concentrated with their titers expressed as viral particles (VPs), as previously described^9^,^18^.

### Induction of CIA and isolation of SFs

Male 8-week old DBA/1(J) mice housed under the specific pathogen-free condition, were immunized intradermally with bovine type II collagen 100 μg in 50 μl 0.1 M acetic acid (Elastin Products) emulsified with 50 μl Freund’s complete adjuvant (4 mg/ml, Chondrex) at the tail base on day 0, and received the intraperitoneal booster of bovine type II collagen 100 μg in 50 μl 0.1 M acetic acid without adjuvant on day 21, resulting in a more than 90% incidence of arthritis, as previously reported^18^,^19^. SFs isolated from CIA synovial tissues and cultured continuously until confluence, were used for further experiments with lines between the 4^th^ and 7^th^ passage.

### Delivery of lentiviral vectors and evaluation of arthritis

On day 36 during the progression of arthritis, CIA mice received i.a. injections of 1 × 10^9^ VPs of LVSmad7 and LVnull into right and left ankle joints, respectively, with medium injection alone as another control group. Arthritis severity was scored on a 0 to 4 scale in each posterior paw with 0: no evidence of erythema and swelling, 1: erythema and mild swelling confined to the tarsals or ankle joint, 2: erythema and mild swelling extending from the ankle to the tarsals, 3: erythema and moderate swelling extending from the ankle to metatarsal joints, and 4: erythema and severe swelling encompassing the ankle, foot and digits, or ankylosis of the limb, as previously described^18^,^20^. Hematoxylin and eosin (H&E)-stained paraffin-embedded ankle joint sections were evaluated for synovial hyperplasia, cartilage erosion, and inflammatory cell infiltration, and a histologic score of 0–2 scale was assigned for each of these features (0: absent, 1: mild, 2: severe) with maximum of 6, as previously described^18^,^19^. Synovial immunohistochemical staining was performed with anti-Smad7 (R&D systems), anti-phosphorylated Smad2/3 (Santa Cruz), anti-IL-17 (eBioscience), anti-TNF (Santa Cruz) or isotype control IgG (Santa Cruz) with the expression intensities quantified by HistoQuest software (TissueGnostics).

### Immunoblot and immunoprecipitaion assessment

Cell lysates of SFs or synovium homogenates were subjected to immunoblot with anti-Smad7 (R&D systems), anti-Flag (Sigma-Aldrich), anti-Snail (Cell Signaling), anti-Cadherin-11 (Cell Signaling), anti-acetylated-histone 3 (Cell Signaling) or anti-β-actin (Sigma-Aldrich), followed by secondary antibodies, developed with ECL Plus system (Amersham), analyzed by Biospectrum imaging system (UVP), and quantitated for the signaling intensities by ImageJ software (National Institutes of Health), as previously described^9^,^18^. Expression of acetylated Smad7 in TSA (Sigma-Aldrich)-treated SFs lysates was immunoprecipitated by anti-Smad7 (Santa Cruz), followed by anti-acetylated-lysine (Cell Signaling) immunoblot with small quantities (~10%) snapped for immunoblot analysis as an input control.

### Statistical analysis

Data are expressed as the mean ± SEM. Immunohistochemical intensities between RA and OA patients were compared by the Mann-Whitney U test. Differences in arthritis scores were calculated by the repeated-measures analysis of variance. Other data was assessed with the Student’s *t*-test. *P* values less than 0.05 were considered significant.

## Additional Information

**How to cite this article**: Chen, S.-Y. *et al*. Intraarticular overexpression of Smad7 ameliorates experimental arthritis. *Sci. Rep.* **6**, 35163; doi: 10.1038/srep35163 (2016).
